# Exploring the Link among State of Mind Concerning Childhood Attachment, Attachment in Close Relationships, Parental Bonding, and Psychopathological Symptoms in Substance Users

**DOI:** 10.3389/fpsyg.2016.01193

**Published:** 2016-08-09

**Authors:** Alessandro Musetti, Grazia Terrone, Paola Corsano, Barbara Magnani, Sergio Salvatore

**Affiliations:** ^1^Department of Literature, Arts, History and Society, University of ParmaParma, Italy; ^2^Department of Humanities, Literature, Cultural Heritage, University of FoggiaFoggia, Italy; ^3^Private Practitioner, Reggio EmiliaItaly; ^4^Department of History, Society and Human Studies, University of SalentoLecce, Italy

**Keywords:** attachment, parental bonding, drug-addiction, psychopathology, paranoid ideation

## Abstract

**Background:** In the present study, we have explored the link among styles of attachment and psychopathology in drug users. We know that insecure attachment predisposes the individuals the development of drug-addiction and psychopathological symptoms. However, we do not know which attachment is more frequent in drug users and which is related to particular psychopathological symptoms. The aim of the present work is to explore the relationship between childhood attachment state of mind, attachment in close relationships, parental bonding and psychopathology in sample of Italian substance users.

**Methods:** We explored, in a sample of 70 drug users and drug-addicted patients, the childhood attachment state of mind measured by the Adult Attachment Interview, the attachment in close relationships by the Relationship Questionnaire and parental bonding measured by the Parental Bonding Instrument. The Symptom Check-List-90-R (SCL-90-R) measured psychopathological symptoms.

**Results:** We found that parental bonding, rather than state of mind concerning childhood attachment or attachment in close relationships, is related to the psychopathological manifestation of anxiety, hostility, depression, and paranoid ideation in the sample. The latter occurs frequently in our sample, independent of state of mind concerning child attachment, attachment in close relationships, and parental bonding, suggesting its role either as a factor that favors a bad image of the participants’ own relationships or as a direct effect of consuming drugs.

**Conclusion:** These results have clinical implications on suggesting ways of interventions that prevent drug-addiction, which should include the evaluation of attachment in the prodromic phases of substance use onset or rehabilitation programs to prevent and manage psychotic-like symptoms.

## Introduction

Main theories consider drug-addiction as an attempt to self-medicate emotional distress (Newcomb, 1995), to cope with “emotional instability and lack of control” (Petraitis et al., 1998) and an overall affective, cognitive, and behavioral dysregulation (Dawes et al., 2000; Sullivan and Farrell, 2002; Corsano et al., 2014). It is expressed within relationships (Schindler et al., 2007, 2009). The ability to regulate distress and to cope with negative feelings in relationships is learnt through attachment. Bowlby (1969, 1973) defined attachment as the emotional bond between a child and his or her caregivers, which provides the emotional “secure base” for personality development. Security depends on the availability and responsiveness of the primary attachment figure, usually the mother. Subsequent work by Ainsworth et al. (1978) led to a first classification of types of attachment (secure, anxious-ambivalent and avoidant) that was later extended by Main and Solomon’s (1990) disorganized type. Although insecure attachment certainly does not predict the development of psychopathology, it creates vulnerability due to inflexible maladaptive strategies for interpreting and interacting with the world (Carlson and Sroufe, 1995). To sum up, attachment is a prototype relational structure that organizes emotion-relational regulation and provides antecedents for the development of psychopathology and drug use (Iglesias et al., 2014; Schindler and Bröning, 2015; Terrone et al., in press).

The aim of the present work is to explore the relationship among state of mind concerning childhood attachment, attachment in close relationships in adulthood, parental bonding, and psychopathology in a clinical sample of Italian drug users. We searched for the most common attachment in the clinical sample and for the most commonly expressed pattern of psychopathological symptoms.

Many studies dealt with attachment and drug use but the results are partly discordant. A link between fearful attachment and addictive disorders has been repeatedly demonstrated (Hazan and Shaver, 1987; Fonagy et al., 1996; Schindler et al., 2007; Piehler et al., 2012). In a study on adults with a long history of drug abuse, 61% of the participants classified themselves as avoidant and only 12% as anxious/ambivalent (Finzi-Dottan et al., 2003). In other studies on opiate addicts, fearful attachment was the predominant style (Schindler et al., 2005, 2009). Molnar et al. (2010) found a relationship between anxious attachment and greater alcohol use in youths aged 19, whereas the relationship between drinking and avoidant attachment appeared to be mediated by social and affective factors.

With regards to attachment and psychopathological symptoms, there is evidence that preoccupied adults manifest passive dependence, confusion, and anger (Main and Goldwyn, 1998, unpublished). Empirical studies suggest a link between preoccupied attachment, anxiety symptoms and borderline personality (Kobak and Sceery, 1988; Patrick et al., 1994; Cole-Detke and Kobak, 1996; Fonagy et al., 1996; Pianta and Walsh, 1996; Rosenstein and Horowitz, 1996). Self-reported preoccupied attachment has been associated with high neuroticism and low self-control (Allen et al., 1998). By contrast, adults with dismissing attachment have childhood histories of avoidant attachment; they reach distance, control, and independence (Shaver and Mikulincer, 2002) and they develop perfectionism, anger, denial, narcissism, and paranoia (Bowlby, 1973; Carlson and Sroufe, 1995; Rosenstein and Horowitz, 1996; Dozier et al., 2001).

From this exploration of the literature on attachment, drug use and psychopathology, a strong link emerges between the three variables. There is confusion about definitions (that is perhaps dependent on the instruments used to measure the construct) and conceptualization of attachment (childhood or adult attachment), the substance addiction considered and the conceptualization of psychopathology. Moreover, attachment studies are usually cross-sectional (Rosenstein and Horowitz, 1996), so we cannot determine how insecure attachment in childhood influences attachment and psychopathology in adulthood and, more importantly, which aspect of the construct of attachment is more explicative of psychopathology in adult drug users. Given these theoretical and methodological issues, we decided to measure attachment with different instruments based on different constructs and methodological conceptualizations. We used the Adult Attachment Interview (AAI – George et al., 1985, unpublished), a semi-structured interview to classify an adult’s state of mind regarding attachment. In the interview, participants describe their relationships to caregivers mainly during childhood by recounting specific memories. They talk about events activating the attachment system, such as separations from caregivers, any losses or trauma and about the effects of childhood experiences on their current personalities. With the Relationship Questionnaire (RQ – Bartholomew and Horowitz, 1991) we looked for the adult attachment in close relationships as a self-reported measure. Finally, with the Parental Bonding Instrument (PBI – Parker et al., 1979) we measured the parental bonding perceived by our adult participants as they remember it up to 16 years of age, once again as a self-reported measure. The AAI instrument is grounded in the first “subculture” of attachment studies (e.g., Bretherton, Cassidy, Crittenden, Kobak, Main, and Waters). They tend to think psychodynamically about clinical problems, they prefer interview measures and behavioral observations over questionnaires, their studies have relatively small groups of subjects, and they focus their attention on parent–child relationships. The self-report questionnaire, by contrast, belongs to a tradition of personality and social psychologists who tend to think about personality traits and social interactions. They prefer simple questionnaire measures to study relatively large samples and focus on adult social relationships, including friendships, dating relationships, and marriages (Bartholomew and Shaver, 1998). Therefore, we can consider the attachment from AAI as a general stable asset for the development of personality while the attachment from RQ and PBI as a more flexible perception depending on the participants’ current emotional or psychopathological state.

What we know from the literature about the relationship between attachment and drugs is that parental bonding, rather than the state of mind concerning childhood attachment, (Kerns et al., 2001) can clearly distinguish between addicted and non-addicted individuals. Moreover, a study found that optimal parental bonding is a strong protective factor that prevents drug use in adolescents (Kostelecky, 2005). Specifically, drug-addicted patients showed affectionless control and affectionate constraint parental bonding (Segura-García et al., 2016).

In the present work, which further explores the state of mind concerning childhood attachment, attachment in close relationships and parental bonding, we have also focused on psychopathological symptoms rather than specific diagnosis and the type of substance used. We have included in the sample adult patients with a diagnosis of drug use or drug-addiction associated or not with a psychiatric disorder.

We expect that the state of mind concerning childhood attachment, in our clinical sample, is mainly insecure and we predict a lack of a relationship between state of mind concerning childhood attachment and a pattern of psychopathological symptoms. Since the self-report instruments (RQ and PBI) are more sensitive to the current expression of relationships, we should find a more explicative association between attachment in close relationships and parental bonding with psychopathological symptoms. Finally, we predict that affectionless control and affectionate constraint will be the most common type of parental bonding in our clinical population.

## Materials and Methods

### Participants

Seventy patients (54 male; mean age = 28.9 years; *SD* = 5.7 years) under treatment for drug-related problems from the public services of Northern Italy (Public Service for Drug and Alcohol Addiction SERT – Servizio per le Tossicodipendenze, for out-patients, or Community, for in-patients) were recruited in the study. The patients were diagnosed by expert clinicians who referred to DSM-5 taxonomy. The inclusion criteria were a diagnosis of substance-use (non-pathological use) or substance-addiction (pathological use as intended by DSM definition of addiction) in association or not with a personality disorder (see **Table 1** for descriptive analysis of the sample). The majority of the participants reported the use of multiple drugs with cannabinoid as primary substance and heroine and/or cocaine as a secondary drug. About 56% of the participants had voluntary access to services while the other 54% was referred by medical doctors, family or other services. Interestingly, 97% of our participants were inserted in a therapeutic program independently of whether they were in-patients or out-patients.

**Table 1:** Number of patients for all categorial variables (gender; public services; diagnosis; AAI-groups; RQ-groups; PBI-groups).

	Number of patients
**Gender**	
Male	57
Female	13
**Public services**	
In-patients	16
Out-patients	51
**Diagnosis**	
Drug use	47
Drug-addiction	9
Drug use and personality disorder	2
Drug-addiction and personality disorder	9
**AAI – groups**	
Secure	32
Dismissing	22
Preoccupied	10
Unresolved loss	3
**RQ – groups**	
Secure	12
Preoccupied	27
Fearful	7
Dismissing	21
**PBI – groups**	
Affectionate constraint	31
Optimal bonding	1
Affectionless control	10
Neglectful parenting	8
Opposite bonding in the two parents	17

All of the patients gave their informed consent to participate in the study.

The study was designed and carried out according to the Ethical Code of the Italian Association of Psychology (AIP) and the American Association of Psychology (APA).

### Instruments

#### Adult Attachment Interview – AAI

The AAI (George et al., 1985, unpublished) is a semi-structured interview of 20 questions that is audio-recorded and transcribed verbatim. The transcript is encoded according to the system formalized by Main et al. (2002). Experts provide scores from 1 to 9 on two groups of scales. Five scales refer to “probable past experiences” (Loving, Rejection, Neglecting, Role Reversal, and Pressure to Achieve), and eleven scales evaluate the “state of mind” with respect to attachment (Idealization, Lack of Memory, Anger, Derogation, Passivity, Transcript Coherence, Mental Coherence, Metacognitive Monitoring, Fear of Loss, Unresolved Loss, Unresolved Trauma). The transcripts are then assigned to one of three principal categories: secure-autonomous (*free-autonomous*, F/A), insecure-distancing (*dismissing*, Ds), insecure-concerned (*enmeshed*, E). There are two additional categories, the unresolved-disorganized relative to loss or trauma (*unresolved*, U) and cannot-classify (*cannot classify*, CC) for unorganized states of mind.

#### Relationship Questionnaire – RQ

The RQ (Bartholomew and Horowitz, 1991) is a questionnaire that measures adult attachment styles. It is composed of 4 sentences describing general relationship styles. Participants are required to indicate how much they disagree or agree with each sentence on a Likert scale from 1 to 7. Using the configuration scores it is possible to classify participants into 4 groups: secure, preoccupied, fearful, dismissing.

#### Parental Bonding Instrument – PBI

The PBI (Parker et al., 1979) is a self-report questionnaire that measures parental styles. It is a ‘retrospective’ measure of how adults (over 16 years) remember their parents during their first 16 years. It is composed of 25 Likert-type items (from 0 to 3) describing both the father’s and the mother’s bonding attitude. Two scores are obtained for each parent: a care and an overprotection score. The care score describes parental attitudes such as love, trust, empathy and warmth (high scores) or emotional coldness, indifference, neglect, and rejection (low scores). The overprotection score describes behaviors and parental attitudes, such as control, intrusiveness, excessive contact and discouragement of independent conduct (high scores) or stimulus of autonomy, freedom, and exploration (low scores). Depending on the intersection of the two orthogonal dimensions of care and overprotection in the two parents, it is possible to classify the participants into 4 groups: affectionate constraint (high degree of care and overprotection), optimal bonding (high degree of care and little control), affectionless control (low degree of care and strong overprotection), neglectful parenting (low degree of care and overprotection). We added a 5th group for participants who obtained opposite patterns in the two parents (for example: highly caring and overprotective father and low caring and overprotective mother). This fifth category has never been used in the literature. We chose to introduce it to give a more significant description of parental bonding that takes into consideration those participants who give extremely different descriptions of their two parents. Some studies show that: maternal and paternal bonding have different roles on personality development (Furnham and Cheng, 2000; Al-Yagon, 2014; Lickenbrock and Braungart-Rieker, 2015); that the mother’s and father’s caregiving styles can have different results in the psychometric scales (Meier et al., 2014); that opposite bonding as measured with the PBI is a factor considered in the etiology of psychopathology (Gao et al., 2010). We then chose to distinguish the participants who describe a similar parental style in both their parents from those who describe an opposite style in order to compare them in the analysis.

For each instrument, descriptive values are reported in **Table 1**.

#### Symptom Checklist-90r – SCL-90-R

The SCL-90-R (Derogatis, 1994) is a 90-item (Likert-type, from 0 to 4) questionnaire assessing nine primary symptom dimensions (somatization, obsessive–compulsive disorder, interpersonal sensitivity, depression, anxiety, hostility, phobic anxiety, paranoid ideation, and psychoticism) and 3 other validity sub-scales.

### Statistical Analysis

We performed a Multivariate Analysis of Variance (MANOVA) to investigate whether the representations of childhood attachment, adult attachment and parental bonding are related to a specific pattern of psychopathological symptoms in individuals with drug-related problems. We considered the nine dimensions of SCL-90-R (somatization, obsessive–compulsive disorder, interpersonal sensitivity, depression, anxiety, hostility, phobic anxiety, paranoid ideation, and psychoticism) as dependent variables and the following ones as independent-categorial variables: public services (in-patients; out-patients); diagnosis (drug use; drug-addiction; drug use and personality disorder; drug-addiction; and personality disorder); AAI-groups (secure, dismissing, preoccupied, unresolved loss); RQ-groups (secure, preoccupied, fearful, dismissing); PBI-groups (affectionate constraint; optimal bonding; affectionless control; neglectful parenting; opposite bonding in the two parents). The variable age was considered as a covariate variable. *Post hoc* analyses were conducted with the Bonferroni-test. Effect size is reported as partial eta-square.

Significant main effects and their pairwise comparisons will be illustrated for each independent-categorial variable. Finally, the result of the covariate variable will be exposed.

## Results

### Public Services

The variable public services resulted significant [*F*_(1,52)_ = 7.11, *p* < 0.05, ∣^2^ = 0.337] for paranoid ideation. Mean values revealed that in-patients presented higher scores in paranoid ideation (4.75) than out-patients in the SERT services (4.46).

### Diagnosis

The variable diagnosis resulted significant [*F*_(3,52)_ = 15.09, *p* < 0.001, ∣^2^ = 0.764) for paranoid ideation. *Post hoc* comparisons showed that a double diagnosis induced higher scores in paranoid ideation (drug use and personality disorder = 0.78; drug-addiction and personality disorder = 0.73) than a single diagnosis (drug use = 0.29, *p* < 0.01; drug addiction = 0.39, *p* < 0.01).

### AAI-groups

The variable AAI-groups resulted significant [*F*_(3,52)_ = 12.31, *p* < 0.001, ∣^2^ = 0.725] for paranoid ideation. However, no *post hoc* comparisons were significant (mean scores: secure = 0.41; dismissing = 0.38; preoccupied = 0.44; unresolved loss = 0.21; *p* > 0.21 for all comparisons).

The variable AAI-groups resulted significant [*F*_(3,52)_ = 24.52, *p* < 0.001, ∣^2^ = 0.840] for the somatization. Surprisingly, *post hoc* comparisons showed that a representation of a secure attachment induced higher scores in somatization (6.10) than a representation of a dismissing (3.48), preoccupied (3.28), and unresolved loss (4.47, *p* < 0.05 for all comparisons).

### RQ-groups

The variable RQ-groups resulted significant [*F*_(3,52)_ = 62.58, *p* < 0.001, ∣^2^ = 0.931] for paranoid ideation. *Post hoc* comparisons showed that a fearful attachment induced higher scores in paranoid ideation (1.05) than a secure (0.01), preoccupied (0.14), or dismissing attachment (0.48, *p* < 0.001 for all comparisons). The variable RQ-groups resulted significant [*F*_(3,52)_ = 3.54, *p* < 0.05, ∣^2^ = 0.431] for the hostility. *Post hoc* comparisons showed that a fearful attachment induced higher scores of hostility (1.73) than a secure (0.83) or a preoccupied attachment (0.57, *p* < 0.05 for all comparisons).

### PBI-groups

**FIGURE 1:**
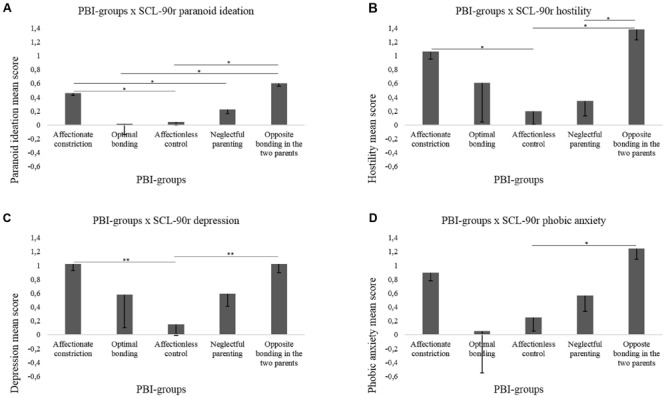
**The figure shows significant interactions between PBI-groups in which patients were classified and the scores in the SCL-90-R dimensions (A - paranoid ideation; B - hostility; C - depression; D - phobic anxiety)**.

Similarly to the other variables, the PBI-groups resulted significant [*F*_(3,52)_ = 6.44, *p* < 0.01, ∣^2^ = 0.580] for paranoid ideation. *Post hoc* revealed that affectionate constraint induced higher scores in paranoid ideation (0.46) than affectionless control (0.04) or neglectful parenting (0.22, *p* < 0.05 for all comparisons, see **Figure 1A**). Opposite bonding in the two parents induced higher scores in paranoid ideation (0.60) than affectionless control (0.04) or optimal bonding (0.02, *p* < 0.05 for all comparisons). In line with the variable RQ-groups, the variable PBI-groups resulted significant [*F*_(3,52)_ = 4.65, *p* < 0.05, ∣^2^ = 0.499] for hostility. *Post hoc* revealed that affectionate constraint induced higher scores in hostility (1.06) than affectionless control (0.20, *p* < 0.05, see **Figure 1B**). Opposite bonding in the two parents induced higher scores in hostility (1.38) than affectionless control or neglectful parenting (0.35, *p* < 0.05 for all comparisons). The variable PBI-groups resulted significant [*F*_(3,52)_ = 5.31, *p* < 0.05, ∣^2^ = 0.532] for the SCL-90-R depression scale. *Post hoc* revealed that both affectionate constraint (1.02) and opposite bonding in the two parents (1.02) induced higher scores in depression than affectionless control (0.15, *p* < 0.01 for all comparisons, see **Figure 1C**). Finally, the variable PBI-groups resulted significant [*F*_(3,52)_ = 6.82, *p* < 0.01, ∣^2^ = 0.594] for phobic anxiety. *Post hoc* revealed that opposite bonding in the two parents induced higher scores in phobic anxiety (1.24) than affectionless control (0.25, *p* < 0.05; see **Figure 1D**).

### Age

The variable age did not significantly covariate with other variables.

## Discussion

Our aim was to explore attachment and psychopathological symptoms in a clinical sample of adult Italian drug users. We measured three different characteristics of attachment: state of mind concerning childhood attachment (George et al., 1985, unpublished), attachment in close relationships (Bartholomew and Horowitz, 1991) and parental bonding (Parker et al., 1979). We expected that the state of mind concerning childhood attachment was only indicative of the risk of developing psychopathology and that insecure attachments would be represented more in the sample than the secure pattern. We also expected that the most represented parental bonding patterns would be the affectionless control and the affectionate constraint ones.

Looking at our sample, we note that the secure AAI-group was as numerous as the sum of the three insecure AAI-groups (dismissing, preoccupied, unresolved loss). This surprising result deserves a particular mention. All of the studies on attachment and substance abuse find a direct link between the insecure style and the use of all kinds of drugs (Schindler and Bröning, 2015). Such studies merely demonstrate a lower consummation of drugs (Branstetter et al., 2009; Danielsson et al., 2012) and a lower risk of developing an addiction pathology but not an absent risk. One explanation for our result is that secure attachment is not causally linked to complete abstinence. Currently, we learn to handle culturally accepted substances (Segura-García et al., 2016) without the need to be predisposed to having a premorbid personality disorders. Individuals with secure childhood attachment can choose to take drugs and perpetuate this behavior because of maintenance factors that occur later in life and do not depend on primary attachment representation. It is worth mentioning that our sample was comprised of drug users and not only drug-addicted patients.

A result immediately linked with the latter is that the secure AAI-group showed higher scores in the somatization SCL-90-R scale. There is no literature about secure AAI-group and somatization, maybe due to the tendency of psychopathology research of focusing on insecure attachment. This result should be taken into consideration carefully, due to the unexpected distribution of secure and insecure attachment in our sample, but it represents an original finding that could elicit new research. It is possible that their worry about health means that they care about themselves even during drug use. A healthy attachment history might provide the asset to relate to somatic symptoms during treatment, so that they care enough to stop using drugs and get their somatic worries to a more healthy level.

In line with our expectations, the analysis revealed that AAI-groups were not related to a particular pattern of psychopathological symptoms, except for a slight relationship with the paranoid ideation symptom that is not significant enough to be discussed in terms of differences among insecure groups. The unforeseen results on AAI-groups support our position to consider other measures of attachment for a chronic disorder such as drug use.

According to our predictions, adult attachment as measured with the RQ explained psychopathological symptoms from the SCL-90-R scales better than the state of mind concerning childhood attachment. Preoccupied and dismissing attachment are related to paranoid ideation and fearful attachment is related to hostility. Other works have used the RQ to measure attachment rather than AAI. Korver-Nieberg et al. (2015) found, in a sample of psychotic inpatients, that preoccupied and fearful attachment was related to severe diagnosis and positive symptoms such as suspiciousness/persecution, excitement, and affective symptoms. They also found that dismissing attachment predisposes individuals to negative symptoms such as social withdrawal. These results are partially in agreement with ours. We can support the notion that anxious attachment (preoccupied and fearful) individuals tend to under-regulate their affection and to show distress. It is not surprising that preoccupied and fearful attachments are most associated with cluster B and especially borderline personality disorder (Timmerman and Emmelkamp, 2006). Preoccupied and fearful attachments are conceptualized as an over-involvement in relationships (Bartholomew and Horowitz, 1991). The preoccupied attachment prompts a dependence on acceptance from others and a tendency to idealize other people. This kind of bond induces patients to pay selective attention to other people’s behavior and emotional expressions in order to have spasmodic confirmation of their acceptance, developing paranoid ideation. Instead, fearful attachment prompts an effort to remain distant in close relationships because of the fear of rejection. This kind of bond induces patients to reject and distrust people, developing hostility. Concerning dismissing attachment, our results are not in agreement with Korver-Nieberg et al. (2015) since we should have found negative symptoms such as depression. It is important to note that Korver-Nieberg et al. (2015) tested psychotic patients, whereas here we have tested drug users. It is possible that the negative symptoms in their sample were more severe than the negative symptoms in our sample.

With regards to parental bonding, we found that the more highly represented groups in our sample were affectionate constraint, affectionless control and opposite patterns of bonding in the two parents. The strong representation of affectionate constraint and affectionless control is in agreement with the literature (Segura-García et al., 2016) and with our expectations.

A useful result for clinical implications is that parental bonding patterns are related to psychopathological symptoms rather than the state of mind concerning childhood attachment and attachment in close relationships. Affectionate constraint and opposite bonding in the two parents are related to anxiety, depression, hostility, and paranoid ideation relative to other types of parental bonding. The evidence underlines a link between “anxiety” bonding and different patterns in the two parents and the development of anxiety disorders (Silove et al., 1991; Picardi et al., 2013). Our group “opposite bonding in the two parents” presented a combination of high care by the mother and low care by the father. In accordance with our results there is evidence that high maternal care, independent of the paternal care style, predicts high levels of anxiety traits but not depressive traits (Parker, 1979). On the other hand, low paternal care is associated with a high level of depression in adolescents (Martin and Waite, 1994). Most importantly, there is evidence of a strong role of low paternal involvement in the development of the affective components (anxiety, anger, depression, and tension) of psychopathy (Farrington, 2006).

Other evidence underlines a relationship between affectionate constraint and extreme personality traits and general severe psychopathology (Amianto et al., 2015). Affectionate constraint is characterized by high care and an overprotective parental style that reduces children’s exploration in the world, which is represented as “dangerous”. It is strongly associated with early expressions of panic disorder (Silove et al., 1991) and severe early obesity (Hancock et al., 2014). There is not much evidence on the affectionate constraint parental style and drug use. However, the latter results suggest that this parental bonding produces a threatening representation of the world with no instruments to cope with it, compatible with feelings of fear, sadness and impotence, distance in relationships and a paranoid interpretation of others’ behavior that can be managed with substances.

One may wonder why neglectful parental bonding is not related with any psychopathological scale. Following Schimmenti (2016), a lack of care by the parents is a strong predictor for the development of dissociation since it interferes with the growth of mentalizing (Schimmenti and Caretti, 2016) and theory of mind processing. It is possible that such participants present meta-cognitive deficits in identifying their perception of their own childhood parental style or their psychological state.

So far, we have discussed our results on our self-report (PBI and RQ) and SCL-90 scales in terms of the influence of parental styles as described in adulthood on personality and psychopathology. Our experimental design does not allow us to speak about a causal influence from the self-report categorization of attachment and expression of symptoms but to only speak about an association between them. Another possible discussion of our results should consider that the way the participants describe their security in relationships could depend on their mood and symptomatology at the moment of compilation. The use of or addiction to multiple drugs, access to a public service or the pathology itself may have induced the participants to feel threatened in their current relationships and to judge them as insecure. This view is supported by Bowlby’s theory stating that insecurity comes out in interpersonal relationships. A person with a state of mind of childhood attachment as secure may feel insecure in a particular moment of his/her life relative to the quality of his/her relationships. It seems that the AAI measure of attachment works as a general interpersonal asset, while the self-report measures (PBI, RQ) underline the diathesis in actual relationships that is more influenced by an attachment-related-threat (Roisman et al., 2007). With regards to the PBI, there is little evidence that its results remain stable over time independent of mood or state of mind at compilation (Martin and Waite, 1994). On the other hand, our result that more severe patients (in-patients and double diagnosis patients) show high levels of paranoid ideation suggests an association between the participants’ degree of impairment and psychotic-like symptoms that could have induced alterations in their state of mind while compiling the self-reports. Further studies are needed to clarify the directionality of the relationship between self-report attachment questionnaires and psychopathology.

In trying to summarize, we found that different aspects of attachment are related differently to psychopathological symptoms in drug problems. This non-linear relationship between attachment and psychopathology is in line with the literature that addresses the issue from a psychoanalytic multifactorial perspective (Barrocas et al., 2016). The common element is the relationship with paranoid ideation. It was more common in the in-patients rather than in the out-patients and in double diagnosis against single diagnosis patients, indicating that this symptom is peculiar to severe addictions. Numerous studies have found associations between addiction disorders and paranoid ideation (Potik et al., 2014; Moss et al., 2015; Del Pino-Gutiérrez et al., 2016) either in terms of vulnerability (Potik et al., 2014) or in terms of the causal effect of drugs on paranoia symptoms. It has been widely demonstrated that all kind of substances, such as cannabis (Freeman et al., 2014) cocaine (Roncero et al., 2014), methamphetamine (Kalayasiri et al., 2014), and alcohol (Bouzyk-Szutkiewicz et al., 2012), induce paranoid ideation. All drugs are associated with an impairment of the dopaminergic system with a direct alteration of striatal functioning for stimulating substances (Milella et al., 2016) or an indirect effect on inhibitory systems (Colizzi et al., 2016). What is important here is that the drugs exacerbate psychotic-like symptoms independent of the kind of substance and the personality of the individuals. This position is supported by our data, which show that insecure attachment predisposes to paranoid ideation and that this symptom is significantly present in severe rather than mild cases.

This study presents some limits. First, we did not collect information concerning the time of drug consumption or the time of treatment that could have provided precious details on the participant’s state of mind at the moment of the experiment. We would have liked to have investigated the issue further in order to draw a picture. Moreover, a control group of non-user participants could have clarified some interpretation of the directionality of the effects. Finally, our analysis considers attachment variables as categorial for all instruments and thus prevents us from making any comparison between potential continuous values on the self-reports (RQ and PBI). Future studies should be implemented following these suggestions.

## Conclusion

Overprotective and opposite parental bonding, rather than child or adult attachment, are explicative of a pattern of psychopathology characterized by anxiety, hostility, depression, and paranoid ideation. The latter is the most characteristic symptom in drug use syndromes, independently of the substance, either as a predisposal factor and/or as a direct effect of consuming drugs. These results have strong research and clinical implications since they suggest we should think about creating studies that better identify the risk of developing substance use by evaluating attachment styles, especially in a current relationship, or to design rehabilitation programs that prevent and manage psychotic-like symptoms in drug users.

## Author Contributions

AM Substantial contributions to the conception of the work, deep analysis of the literature, study design, development, and final approval of the paper. GT Contribute to the development and revision of the work with deep literature analysis, data acquisition, data analysis and agreement for final approval of the paper. PC Contribute to the development and revision of the work, with literature analysis and agreement for final approval of the paper. BM Contribute to the development and revision of the work, contribution to data analysis, and agreement for final approval of the paper. SS Contribute to development and deep revision of the work, with literature analysis and agreement for final approval of the paper.

## Conflict of Interest Statement

The authors declare that the research was conducted in the absence of any commercial or financial relationships that could be construed as a potential conflict of interest.
